# Prevalence of Angina Among Primary Care Patients With Coronary Artery Disease

**DOI:** 10.1001/jamanetworkopen.2021.12800

**Published:** 2021-06-07

**Authors:** Daniel M. Blumenthal, Sidney E. Howard, Jennifer Searl Como, Sandra M. O’Keefe, Steven J. Atlas, Daniel M. Horn, Neil W. Wagle, Jason H. Wasfy, Robert W. Yeh, Joshua P. Metlay

**Affiliations:** 1 Cardiology Division, Department of Medicine, Massachusetts General Hospital, Boston; 2 Harvard Medical School, Boston, Massachusetts; 3 Coeur Value, LLC, Wellesley, Massachusetts; 4 Mongan Institute, Massachusetts General Hospital, Boston; 5 Massachusetts General Physicians Organization, Massachusetts General Hospital, Boston; 6 Division of General Internal Medicine, Department of Medicine, Massachusetts General Hospital, Boston; 7 Devoted Health, Waltham, Massachusetts; 8 Division of General Internal Medicine, Brigham and Women’s Hospital, Boston, Massachusetts; 9 Smith Center for Outcomes Research in Cardiology, Beth Israel Deaconess Medical Center, Boston, Massachusetts; 10 Cardiology Division, Beth Israel Deaconess Medical Center, Boston, Massachusetts

## Abstract

**Importance:**

Angina pectoris is associated with morbidity and mortality. Angina prevalence and frequency among contemporary US populations with coronary artery disease (CAD) remain incompletely defined.

**Objective:**

To ascertain the angina prevalence and frequency among stable outpatients with CAD.

**Design, Setting, and Participants:**

This cross-sectional survey study involved telephone-based administration of the Seattle Angina Questionnaire–7 (SAQ-7) between February 1, 2017, and July 31, 2017, to a nonconvenience sample of adults with established CAD who receive primary care through a large US integrated primary care network. Data analysis was performed from August 2017 to August 2019.

**Exposure:**

SAQ-7 administration.

**Main Outcomes and Measures:**

Angina prevalence and frequency were assessed using SAQ-7 question 2. Covariates associated with angina were assessed in univariable and multivariable regression.

**Results:**

Of 4139 eligible patients, 1612 responded to the survey (response rate, 38.9%). The mean (SD) age of the respondents was 71.8 (11.0) years, 577 (35.8%) were women, 1447 (89.8%) spoke English, 147 (9.1%) spoke Spanish, 1336 (82.8%) were White, 76 (4.7%) were Black, 92 (5.7%) were Hispanic, 974 (60.4%) had Medicare, and 83 (5.2%) had Medicaid. Among respondents, 342 (21.2%) reported experiencing angina at least once monthly; among those, 201 (12.5%) reported daily or weekly angina, and 141 respondents (8.7%) reported monthly angina. The mean (SD) SAQ-7 score was 93.7 (13.7). After multivariable adjustment, speaking a language other than Spanish or English (odds ratio [OR], 5.07; 95% CI, 1.39-18.50), Black race (OR, 2.01; 95% CI, 1.08-3.75), current smoking (OR, 1.88; 95% CI, 1.27-2.78), former smoking (OR, 1.69; 95% CI, 1.13-2.51), atrial fibrillation (OR, 1.52; 95% CI, 1.02-2.26), and chronic obstructive pulmonary disease (OR, 1.61; 95% CI, 1.18-2.18) were associated with more frequent angina. Male sex (OR, 0.63; 95% CI, 0.47-0.86), peripheral artery disease (OR, 0.63; 95% CI, 0.44-0.90), and novel oral anticoagulant use (OR, 0.19; 95% CI, 0.08-0.48) were associated with less frequent angina.

**Conclusions and Relevance:**

Among stable outpatients with CAD receiving primary care through an integrated primary care network, 21.2% of surveyed patients reported experiencing angina at least once monthly. Several objective demographic and clinical characteristics were associated with angina frequency. Proactive assessment of angina symptoms using validated assessment tools and estimation of patients at higher risk of suboptimally controlled angina may be associated with reduced morbidity.

## Key Points

**Question:**

What is the prevalence of angina among stable US outpatients with coronary artery disease (CAD)?

**Findings:**

In a survey study of 1612 outpatient primary care patients with CAD in a large US integrated primary care network, 21.2% of surveyed patients reported experiencing angina at least once per month (daily or weekly, 12.5%; monthly, 8.7%). After multivariable adjustment, speaking a language other than Spanish or English, Black race, smoking, atrial fibrillation, and chronic obstructive pulmonary disease were associated with increased angina frequency.

**Meaning:**

These findings suggest that angina is prevalent among US outpatients with CAD; proactive angina assessment in outpatient settings may identify patients with suboptimally controlled angina and may be associated with improved treatment and outcomes.

## Teaser

This survey study examines the prevalence and frequency of angina among stable US outpatients with coronary artery disease.

## Introduction

Heart disease is the most common cause of death in the US. Coronary artery disease (CAD) causes the majority of deaths due to heart disease and accounts for approximately 1 in 7 US deaths each year.^1^ Angina pectoris (angina) is a common symptom of obstructive CAD and is associated with major adverse cardiovascular events, reduced quality of life, and higher costs of care.^2,3^

Prior work has characterized the epidemiology of angina among patients with newly diagnosed obstructive CAD or who have recently experienced myocardial infarction or undergone revascularization. Less work has investigated the prevalence, severity, and clinical consequences of angina among patients with medically managed chronic CAD, including patients with both obstructive and nonobstructive CAD and those cared for by primary care physicians (PCPs), as opposed to cardiologists.^2,4,5,6,7,8,9,10^ Previous investigations of angina prevalence in primary care may have limited generalizability to contemporary US primary care populations for one of several reasons, including that they are outdated or focused on veterans, patients with a history of anginal symptoms, or international populations.^11,12,13,14,15,16,17^

To address these limitations, we administered the Seattle Angina Questionnaire (SAQ)–7 by telephone to a sample of outpatients with diagnosed CAD who receive care within a large primary care network that is part of an integrated health care delivery system.^1,18,19^ The goals of this study were to (1) characterize angina frequency among all patients with CAD who are part of a large primary care community and (2) identify covariates associated with angina frequency in this patient population.

## Methods

We conducted a cross-sectional survey of a primary care clinic–based sample of adults with established CAD. This study was approved by the Mass General Brigham institutional review board, which also approved a waiver for informed consent because the data were deidentified, in accordance with 45 CFR §46. Survey eligibility disposition status and survey completion rates were categorized and reported in accordance with the American Association for Public Opinion Research (AAPOR) standard definitions for telephone survey eligibility (eMethods in the Supplement).^20^

### Seattle Angina Questionnaire–7

The SAQ-7 is a validated 7 question survey instrument derived from the original SAQ, an extensively validated 19-item questionnaire designed to assess the health status of patients with CAD.^18^ The SAQ-7 assesses 3 dimensions of health among patients with CAD: physical limitation, angina frequency, and quality of life (eMethods in the Supplement).^1^

### Study Population

We sampled patients from 15 primary care clinics and community health centers that are part of the Massachusetts General Hospital (MGH) Primary Care Practice Based Research Network (PBRN). These 15 sites span urban and suburban sites and are staffed by MGH-employed PCPs. We used PBRN data to develop a comprehensive list of MGH primary care patients with CAD. The PBRN contains insurance claims and electronic health record (EHR) data for 161 000 MGH primary care patients.

Patients with CAD aged 30 years or older were eligible for study participation. Eligible patients were identified using a validated, electronic algorithm that uses both billing claims information—specifically, *International Classification of Diseases, Ninth Revision *(*ICD-9*) or *International Statistical Classification of Diseases and Related Health Problems, Tenth Revision *(*ICD-10*) codes—and EHR information, such as inclusion of CAD, coronary revascularization, or myocardial infarction, as a problem on a patient’s problem list, to identify patients with CAD (inclusion criteria definitions are shown in eMethods in the Supplement). Importantly, patients with asymptomatic and nonobstructive CAD were eligible for inclusion. Compared with manual medical record review (eg, the reference standard), the search algorithm identified patients with CAD with sensitivity greater than 90% and specificity of 90%.^21^

Presurvey ineligibility criteria included a history of moderate-to-severe dementia documented in the EHR, which was ascertained by population health coordinators (PHCs) employed by the Massachusetts General Physicians’ Organization, who screened patients’ medical records before administering surveys. Patients were also deemed ineligible before or at the time of survey administration if they were no longer seeing an MGH-employed PCP, were hospitalized, in hospice, or in a nursing home.^19^

### Participant Selection

At the outset, we stratified the study sample by clinical practice to survey representative proportions of patients from different practices. Within each practice, survey participants were sampled randomly, with the goal of surveying at least 50 patients per practice. Funding constraints prevented us from surveying all patients with CAD in the PBRN. We planned an interim comparison of surveyed and nonsurveyed populations to identify racial, ethnic, or socioeconomic subpopulations that were underrepresented among survey respondents and to prioritize surveying underrepresented groups. This analysis was performed during June 2017 and focused on race/ethnicity and median household income by US Census tract.

### Survey Administration

SAQ-7 administration was overseen by the Massachusetts General Physicians’ Organization as part of a quality improvement project. Survey administration methods have been previously described and published.^19^ Surveys were administered by telephone (and in rare circumstances via secure email per patient preference) between February 1, 2017, and July 31, 2017, by PHCs. The Massachusetts General Physicians’ Organization employs 7 full-time PHCs, who are lay professionals and routinely contact and coordinate testing and appointments for patients enrolled in MGH practices. PHCs administered the SAQ-7 while doing their normal daily telephone contact of primary care patients. One PHC was fluent in Spanish and was preferentially assigned responsibility for surveying Spanish-speaking patients. We drafted scripts and protocols for contacting patients, administering surveys, and recording survey responses in the EHR to standardize these core processes. We conducted two 1-hour training sessions for PHCs focused on signs, symptoms, and pathophysiology of CAD, the study purpose and goals, SAQ-7 administration, and scripts, protocols, and follow-up methods (eMethods in the Supplement).

### Baseline Demographic and Clinical Data

We obtained baseline demographic and clinical characteristics and measures of outpatient clinic access in the 12 months preceding survey administration from the PBRN. Median household income was estimated with median household income by Census tract from the 2010 US Census.

Race and ethnicity were classified using data from patients’ EHRs, which represent patients’ self-reported race and ethnicity. Race and ethnicity were evaluated as covariates to assess for racial and ethnic disparities in angina prevalence.

### Statistical Analysis

First, we compared baseline characteristics of survey respondents and nonrespondents to assess for differences that could suggest selection bias. We next summarized the survey responses, including mean (SD) SAQ-7 score and the frequency distributions across each survey question. We analyzed unadjusted associations between angina symptom frequency—ascertained using responses to SAQ-7 question 2—baseline demographic and clinical characteristics, and health care access patterns before survey administration. We stratified responders into 3 groups: daily or weekly angina (merged because of the small sample size of patients reporting daily angina), monthly angina, or no angina. We compared these 3 groups in terms of their demographic and clinical characteristics and their access to health care before the start of this study (a complete covariate list is provided in eMethods in the Supplement). We calculated unadjusted odds ratios (ORs) with 95% CIs and used 2-sided χ^2^ tests to calculate *P* values, with *P* ≤ .05 considered significant.

We also evaluated whether baseline CAD burden was associated with angina frequency as assessed by the SAQ-7. Using administrative data, we developed and added to our set of baseline characteristics a 3-level covariate as follows: group 1 included patients with a history of angina. Angina was defined as an *ICD-9* or *ICD-10* code consistent with stable angina billed in the 3 years before study enrollment. Group 2 consisted of patients with clinical CAD—defined as an *ICD-9* or *ICD-10* code or EHR problem indicating ischemic heart disease (ie, obstructive CAD), prior myocardial infarction or unstable angina, or prior coronary revascularization—but no angina. Group 3 included all remaining patients with CAD who did not meet criteria for groups 1 or 2 (ie, nonobstructive CAD). We used analysis of variance and 2-sided χ^2^ tests for comparisons of continuous and categorical variables, respectively.

Then we estimated a multivariable multinomial logistic regression model that included 4 covariate categories: demographic covariates, clinical covariates other than medications, medications, and baseline CAD burden (characterized using the 3-level covariate derived from EHR and claims data described already). The model included covariates associated with angina frequency in unadjusted analyses at a threshold of *P* ≤ .10. Dependent variable categories included daily or weekly angina, monthly angina, and no angina.

All analyses were performed in SAS statistical software version 9.4 (SAS Institute). Data analysis was performed from August 2017 to August 2019.

## Results

**Figure 1.**
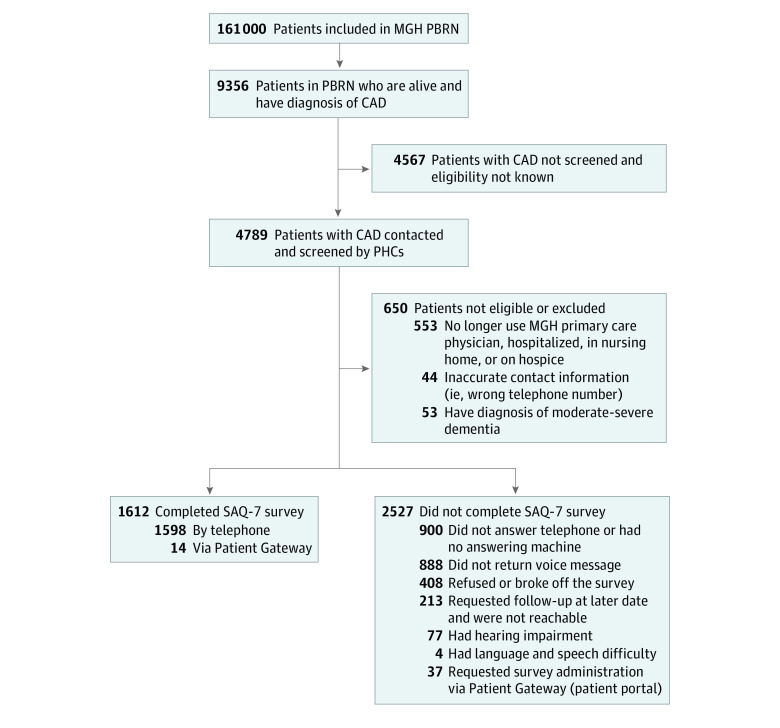
Patient Enrollment Flowchart CAD indicates coronary artery disease; MGH, Massachusetts General Hospital; PBRN, Primary Care Practice Based Research Network; PHC, population health coordinator; SAQ-7, Seattle Angina Questionnaire–7.

The PBRN electronic search algorithm identified 9356 patients with CAD aged 30 years or older who were alive as of June 30, 2016. Of these, PHCs screened 4789 of 9356 patients (51.0%), of whom 4139 of 4789 (86.4%) were deemed eligible for survey completion and were contacted to complete a survey; 650 of 4789 (14.3%) were excluded or deemed ineligible (Figure 1 and eTable 1 in the Supplement).^19^ Of those eligible, 1612 of 4139 (38.9%) completed a survey. Respondents’ mean (SD) age was 71.8 (11.0) years, 577 (35.8%) were women, 1447 (89.8%) spoke English, 147 (9.1%) spoke Spanish, 1336 (82.8%) were White, 76 (4.7%) were Black, 92 (5.7%) were Hispanic, 974 (60.4%) were insured by Medicare, 545 (33.8%) were insured by commercial carriers, and 83 (5.2%) were insured by Medicaid (eTable 2 in the Supplement).

Prespecified interim comparisons of surveyed and nonsurveyed patients did not reveal substantial underrepresentation of any specific prespecified subgroup. Similarly, comparisons of respondents and nonrespondents following study completion showed that respondents were younger than nonrespondents (mean [SD] age, 71.8 [11.0] vs 73.2 [11.2] years; difference, 1.4 years; 95% CI, −2.09 to −0.70 years; *P* < .001), more likely to speak Spanish (9.1% [147 respondents] vs 5.3% [168 nonrespondents]; χ^2^_2_ = 53.73; *P* < .001), and more likely to have Medicaid insurance (5.2% [83 respondents] vs 4.2% [132 nonrespondents]; χ^2^_3_ = 7.80; *P* = .05). Respondents were also more likely to have experienced a history of acute myocardial infarction (12.3% [199 respondents] vs 9.9% [314 nonrespondents]; χ^2^_0_ = 6.77; *P* = .01) (eTable 2 in the Supplement).

**Figure 2.**
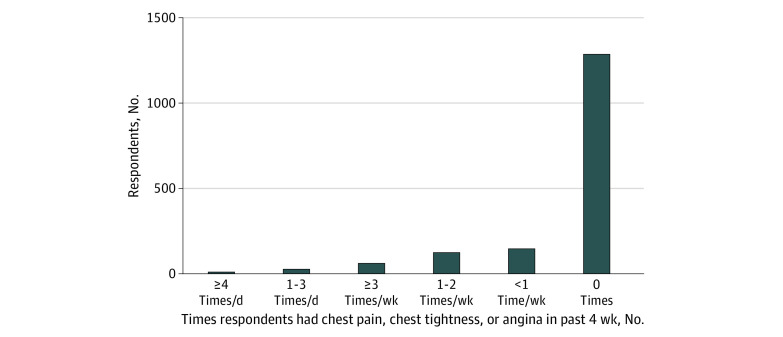
Frequency of Angina Among Seattle Angina Questionnaire–7 Respondents

The mean (SD) total SAQ-7 score was 93.7 (13.7); 1270 respondents (78.8%) reported no angina and 342 (21.2%) reported experiencing angina at least once monthly, with 141 respondents (8.7%) reporting monthly, but not daily or weekly, angina, and 201 respondents (12.5%) reporting daily or weekly angina (Table 1 and Figure 2). The mean (SD) participant age was 72.7 (11.0) years among patients with no angina, 69.8 (10.9) years among those with monthly angina, and 67.7 (10.7) years in patients with daily or weekly angina (*P* for trend, <.001). Of note, if 0% of nonrespondents reported angina, the total population prevalence of angina in the study cohort would be 8.3% (4.9% with daily or weekly angina, and 3.4% with monthly angina).

**Table 1.** Patient Characteristics, Overall and Stratified by Angina Frequency

Variable	Respondents, No. (%)				OR (95% CI)
	All patients (N = 1612)	No angina (n = 1270)	Monthly angina (n = 141)	Daily or weekly angina (n = 201)	
Indication for study participation					
Billing claim consistent with prior angina	230 (14.3)	162 (12.8)	20 (14.2)	48 (23.9)	1 [Reference]
Clinical CAD but no angina	766 (47.5)	624 (49.1)	64 (45.4)	78 (38.8)	0.62 (0.44-0.87)
No clinical CAD	616 (32.2)	484 (38.1)	57 (40.4)	75 (37.3)	0.52 (0.37-0.72)
Demographic characteristics					
Age, mean (SD), y	71.8 (11.0)	72.7 (11.0)	69.8 (10.9)	67.7 (10.7)	0.97 (0.96-0.99)
Sex					
Male	1035 (64.2)	852 (67.1)	80 (56.7)	103 (51.2)	0.56 (0.44-0.71)
Female	567 (35.8)	418 (32.9)	61 (43.3)	98 (48.8)	1 [Reference]
Language					
English	1447 (89.8)	1172 (92.3)	120 (85.1)	155 (77.1)	1 [Reference]
Spanish	147 (9.1)	86 (6.8)	17 (12.1)	44 (21.9)	3.18 (2.26-4.48)
Other^a^	18 (1.1)	12 (0.9)	4 (2.8)	2 (1.0)	1.88 (0.69-5.08)
Race/ethnicity					
White	1336 (82.9)	1089 (85.8)	109 (77.3)	138 (68.7)	1 [Reference]
Black or African American	76 (4.7)	50 (3.9)	8 (5.7)	18 (9.0)	2.38 (1.47-3.86)
Hispanic or Latino	92 (5.7)	52 (4.1)	11 (7.8)	29 (14.4)	3.58 (2.35-5.44)
Other^b^	108 (6.7)	79 (6.2)	13 (9.2)	16 (8.0)	1.60 (1.02-2.49)
Median annual household income, $					
>120 000	243 (15.1)	202 (15.9)	17 (12.1)	24 (11.9)	1 [Reference]
80 001-120 000	403 (25.0)	323 (25.4)	38 (27.0)	42 (20.9)	1.20 (0.79-1.82)
40 001-80 000	637 (39.5)	489 (38.5)	57 (40.4)	91 (45.3)	1.50 (1.02-2.19)
≤40 000	329 (20.4)	256 (20.2)	29 (20.6)	44 (21.9)	1.41 (0.92-2.15)
Insurance type					
Commercial	545 (33.8)	429 (33.8)	45 (31.9)	71 (35.3)	1 [Reference]
Medicaid	83 (5.2)	50 (3.9)	16 (11.4)	17 (8.5)	2.24 (1.39-3.61)
Medicare	974 (60.4)	782 (61.6)	79 (56.0)	113 (56.2)	0.90 (0.70-1.17)
Self	10 (0.6)	9 (0.7)	1 (0.7)	Not applicable	0.38 (0.05-3.21)
Education level					
College graduate	690 (43.7)	567 (45.7)	56 (40.0)	67 (33.8)	1 [Reference]
Some college, vocational, or technical school	168 (10.7)	139 (11.2)	15 (10.7)	14 (7.1)	0.95 (0.61-1.48)
High school or GED	531 (33.7)	397 (32.0)	52 (37.1)	82 (41.4)	1.57 (1.20-2.07)
Some high school but no diploma or GED	99 (6.3)	70 (5.7)	10 (7.1)	19 (9.6)	1.96 (1.23-3.12)
Eighth grade or less	90 (5.6)	67 (5.4)	7 (5.0)	16 (8.1)	1.65 (0.995-2.73)
Primary care clinic affiliation					
Community health center clinic	644 (39.9)	478 (37.6)	66 (46.8)	100 (49.7)	1 [Reference]
Non–health center clinic	968 (60.1)	792 (62.4)	75 (53.2)	101 (50.3)	0.64 (0.50-0.81)
Clinical characteristics					
Smoking status					
Never	409 (30.4)	341 (31.9)	34 (27.3)	35 (22.6)	1 [Reference]
Current	505 (37.6)	378 (35.4)	52 (42.2)	76 (49.0)	1.71 (1.23-2.37)
Former	431 (32.0)	350 (32.7)	37 (30.5)	44 (28.4)	1.17 (0.82-1.66)
Body mass index, mean (SD)^c^	29.7 (5.7)	29.5 (5.6)	30.1 (6.5)	30.3 (6.1)	1.02 (0.99-1.04)
Blood pressure, mean (SD), mm Hg					
Systolic	128 (17)	128 (17)	128 (18)	128 (17)	1.00 (0.99-1.01)
Diastolic	72 (10)	72 (10)	72 (10)	73 (10)	1.01 (1.00-1.02)
Heart rate, mean (SD), beats/min	71 (12)	71 (12)	71 (12)	73 (13)	1.01 (1.00-1.02)
Hemoglobin A_1c_, mean (SD), % of total hemoglobin	6.3 (1.2)	6.3 (1.2)	6.3 (1.1)	6.5 (1.5)	1.10 (0.99-1.23)
Low-density lipoprotein, mean (SD), mg/dL	80 (30.1)	79 (30.4)	82 (34.0)	84 (31.0)	1.004 (1.00-1.01)
Creatinine, mean (SD), mg/dL	1.1 (0.6)	1.2 (0.6)	1.1 (0.5)	1.1 (0.7)	0.87 (0.69-1.10)
Hypertension	1456 (90.3)	1147 (90.3)	128 (90.8)	181 (90.1)	0.99 (0.67-1.49
Diabetes	642 (39.8)	482 (38.0)	65 (46.1)	95 (47.3)	1.43 (1.13-1.82)
Congestive heart failure	471 (29.2)	366 (28.8)	48 (34.0)	57 (28.4)	1.07 (0.83-1.39)
Chronic obstructive pulmonary disease	619 (38.4)	452 (35.6)	59 (41.8)	108 (53.7)	1.77 (1.39-2.25)
Atrial fibrillation	420 (26.1)	339 (26.7)	42 (29.8)	39 (19.4)	0.83 (0.63-1.09)
Prior myocardial infarction	199 (12.3)	154 (12.1)	15 (10.6)	30 (14.9)	0.83 (0.63-1.09)
Peripheral artery disease	407 (25.3)	339 (26.5)	42 (21.3)	41 (20.4)	0.73 (0.55-0.97)
Dementia	79 (4.9)	68 (5.4)	5 (3.6)	6 (3.0)	0.56 (0.31-1.12)
Total Seattle Angina Questionnaire–7 score, mean (SD)	93.7 (13.7)	99.2 (3.4)	84.1 (10.8)	65.5 (16.7)	0.80 (0.79-0.82)
Medication use					
Any statin	1491 (92.5)	1179 (92.8)	130 (92.2)	182 (90.6)	0.79 (0.52-1.21)
High intensity statin	544 (33.8)	407 (32.0)	55 (39.0)	82 (40.8)	1.42 (1.11-1.81)
β-blocker	1269 (78.7)	983 (77.4)	122 (86.5)	164 (81.6)	1.46 (1.07-1.99)
Calcium channel blocker^d^	147 (9.1)	114 (9.0)	14 (9.9)	19 (9.5)	1.8 (0.72-1.62)
Aspirin	1248 (774)	986 (77.6)	106 (75.2)	156 (77.6)	0.95 (0.72-1.26)
Long-acting nitrate	253 (15.7)	166 (13.1)	30 (21.3)	57 (28.4)	2.31 (1.73-3.08)
Short-acting nitrate	617 (38.3)	435 (34.3)	67 (47.5)	115 (57.2)	2.22 (1.74-2.82)
Ranolazine	28 (1.7)	16 (1.3)	3 (2.1)	9 (4.5)	3.04 (1.47-6.30)
P2Y_12_ inhibitor^e^	342 (21.2)	247 (19.5)	33 (23.4)	62 (30.9)	1.63 (1.24-2.13)
Warfarin	216 (13.4)	174 (13.7)	26 (18.4)	16 (8.0)	0.84 (0.58-1.20)
Novel oral anticoagulant^f^	101 (6.3)	95 (7.5)	4 (2.8)	2 (1.0)	0.22 (0.09-0.50)
Insulin	319 (19.8)	238 (18.7)	23 (16.3)	58 (28.9)	1.41 (1.07-1.87)
Health care service use before study initiation					
Outpatient clinic appointments in 2014-2016, mean (SD), No.^g^	20 (12.9)	19 (12.2)	21 (12.6)	24 (16.3)	1.02 (1.01-1.03)
Missed outpatient clinic appointments in 2014-2016, mean (SD), No.^h^	2.2 (1.96)	2.1 (1.9)	2.3 (2.3)	2.5 (2.4)	0.83 (0.47-1.48)
Missed appointment ratio, mean (SD), %^i^	3.8 (7.0)	3.5 (7.0)	5.2 (10.0)	5.0 (8.0)	1.29 (1.12-1.50)
Hospitalized in 2016^j^	294 (18.2)	230 (18.1)	20 (14.2)	44 (21.9)	1.08 (0.80-1.46)

Abbreviations: CAD, coronary artery disease; GED, general equivalency diploma; OR, odds ratio.

SI conversion factors: To convert creatinine to micromoles per liter, multiply by 88.4; hemoglobin A_1c _to proportion of total hemoglobin, multiply by 0.01; low-density lipoprotein to millimoles per liter, multiply by 0.0259.

^a^ Includes all languages other than English or Spanish.

^b^ Includes Native American, Asian, and Pacific Islander.

^c^ Body mass index is calculated as weight in kilograms divided by height in meters squared.

^d^ Includes nondihydropyridine calcium channel blockers.

^e^ Includes clopidogrel, ticagrelor, and prasugrel.

^f^ Includes dabigratran, apixaban, and rivaroxaban.

^g^ Ascertained using billing and appointment data from Partners Healthcare. Does not include clinic appointments with physicians who are not affiliated with Partners Healthcare.

^h^ Ascertained using billing and appointment data from Partners Healthcare. Does not include missed clinic appointments with physicians who are not affiliated with Partners Healthcare.

^i^ The missing appointment ratio is calculated as follows: the number of missed outpatient clinic appointments divided by the total attended and missed outpatient clinic appointments was calculated for each patient. Then, the mean and the SD of these ratios were calculated for each patient subgroup.

^j^ Denotes the proportion of patients with an inpatient admission documented in the electronic health record during 2016. May not include inpatient hospitalizations at facilities outside of Partners Healthcare, the health system which owns the Massachusetts General Hospital.

In unadjusted analyses, angina frequency was lower in men than women (OR, 0.56; 95% CI, 0.44-0.71). Angina frequency was positively associated with speaking Spanish (OR, 3.18; 95% CI, 2.26-4.48), identifying as Black (vs White: OR, 2.38; 95% CI, 1.47-3.86), identifying as Hispanic or Latino (OR, 3.58; 95% CI, 2.35-5.44), having Medicaid insurance (OR, 2.24; 95% CI, 1.39-3.61), completing high school or obtaining a general equivalency diploma (vs college degree, OR, 1.57; 95% CI, 1.20-2.07), and finishing some high school (vs college degree, OR, 1.96; 95% CI, 1.23-3.12). Clinical variables associated with angina frequency included being a current smoker (OR, 1.71; 95% CI, 1.23-2.37), having chronic obstructive pulmonary disease (OR, 1.77; 95% CI, 1.39-2.25), having diabetes (OR, 1.43; 95% CI, 1.13-1.82), and use of a P2Y_12_ inhibitor (OR, 1.63; 95% CI, 1.24-2.13), long-acting nitrates (OR, 2.31; 95% CI, 1.73-3.08), ranolazine (OR, 3.04; 95% CI, 1.47-6.30), or insulin (OR, 1.41; 95% CI, 1.07-1.87). A history of peripheral arterial disease (OR, 0.73; 95% CI, 0.55-0.97) and current use of novel oral anticoagulant (NOAC) (OR, 0.22; 95% CI, 0.09-0.50) were negatively associated with angina frequency. Greater numbers of completed outpatient clinic appointments in the prior year (OR per appointment, 1.02; 95% CI, 1.01-1.03) and a higher missed appointment ratio in the prior year (OR per 10% increase in ratio, 1.29; 95% CI, 1.12-1.50) were also associated with angina frequency.

### Multivariable Regression

In multivariable regression, covariates independently associated with more frequent angina included speaking a language other than Spanish or English (OR, 5.07; 95% CI, 1.39-18.50), Black race (OR, 2.01; 95% CI, 1.08-3.75), current smoking (OR, 1.88; 95% CI, 1.27-2.78), former smoking (OR, 1.69; 95% CI, 1.13-2.51), atrial fibrillation (OR, 1.52; 95% CI, 1.02-2.26), and chronic obstructive pulmonary disease (OR, 1.61; 95% CI, 1.18-2.18) (Table 2). Older age (OR per year of life, 0.97; 95% CI, 0.95-0.98), male sex (OR, 0.63; 95% CI, 0.47-0.86), peripheral artery disease (OR, 0.63; 95% CI, 0.44-0.90), and current NOAC use (OR, 0.19; 95% CI, 0.08-0.48) were associated with less frequent angina. The presence of recent angina or clinical CAD—as assessed using billing claims and EHRs—was not significantly associated with angina frequency in multivariable analysis.

**Table 2.** Demographic, Social, and Clinical Characteristics Associated With Angina Frequency After Multivariable Adjustment^a^

Variable	OR (95% CI)	*P* value^b^
Demographic characteristics		
Age (per year)	0.97 (0.95-0.98)	<.001
Male sex	0.63 (0.47-0.86)	.003
Language		
English	1 [Reference]	
Spanish	2.53 (0.98-6.52)	.06
Other^c^	5.07 (1.39-18.50)	.01
Race		
White	1 [Reference]	.03
Black or African American	2.01 (1.08-3.75)	
Clinical characteristics		
Smoking status		
Never	1 [Reference]	
Current	1.88 (1.27-2.78)	.002
Former	1.69 (1.13-2.51)	.01
Atrial fibrillation	1.52 (1.02-2.26)	.04
Chronic obstructive pulmonary disease	1.61 (1.18-2.18)	.003
Peripheral artery disease	0.63 (0.44-0.90)	.01
Medication use		
Long-acting nitrates	1.52 (1.03-2.23)	.03
Short-acting nitrates	2.67 (1.90-3.74)	<.001
Ranolazine	3.03 (1.17-7.88)	.03
Novel oral anticoagulant^d^	0.19 (0.08-0.48)	<.001

Abbreviation: OR, odds ratio.

^a^ This table presents the results of a multivariable multinomial regression model that included all demographic, social, and clinical covariates associated with angina frequency. Only covariates that were independently associated with angina frequency (at *P* < .05) are presented in this table. The regression model included all covariates associated with angina frequency in univariable analysis at *P* ≤ .10. Angina frequency, the dependent variable, was structured into 3 categories: no angina, monthly angina, and daily or weekly angina.

^b^ *P* values were calculated using χ^2^, analysis of variance, or Kruskal-Wallis tests as appropriate.

^c^ Includes all languages other than English or Spanish.

^d^ Includes dabigratran, apixaban, and rivaroxaban.

## Discussion

In this survey study, we found that 21.2% of patients reported experiencing angina at least monthly, with 12.5% reporting daily or weekly angina and 8.7% reporting monthly angina symptoms. Moreover, after multivariable regression, a history of smoking, atrial fibrillation, and chronic obstructive pulmonary disease were associated with more frequent angina, whereas older age, male sex, and peripheral artery disease were associated with lower angina frequency.

To our knowledge, this study is the first to assess angina prevalence and severity in a nonconvenience sample of stable outpatients with clinical CAD—defined as a history of ischemic heart disease, myocardial infarction, coronary revascularization, or angina—and nonclinical CAD, who have received a diagnosis of CAD but have no history of symptoms or overt clinical manifestations of this disease. Patients without a history of clinically active CAD may still experience disease progression. Understanding the prevalence and severity of angina among all patients with CAD may, therefore, enable more systematic identification of patients with CAD with poorly controlled symptoms.^22,23,24^

Our survey response rate of 38.9% may limit the generalizability of our findings. We also oversampled Spanish-speaking patients, who were more likely to report angina. The prevalence of angina in populations with smaller proportions of Spanish-speaking patients may be slightly below those reported here. We otherwise found only mild and small differences in clinical and demographic characteristics between respondents and nonrespondents but cannot rule out other unmeasured differences. Even so, if 0% of nonrespondents reported angina, the total population prevalence of angina in the study cohort would be 8.3% (4.9% with daily or weekly angina, and 3.4% with monthly angina). This percentage represents a low-end estimate of angina population prevalence among stable outpatients with CAD.

We are aware of no prior studies characterizing clinical and demographic covariates associated with any angina or angina frequency among patients with CAD who receive care through a US primary care network. The prevalence of angina in our study was lower than the rates of 33% to 38% observed in 2 studies^12,25^ characterizing angina prevalence among patients with a history of acute coronary syndrome, coronary revascularization, exercise-induced ischemia on stress testing, or obstructive coronary stenosis. This lower angina rate makes sense conceptually, because our study was not limited to patients with symptomatic or obstructive CAD.

Our findings nonetheless reinforce the substantial prevalence of symptomatic CAD among primary care patients. Understanding this baseline prevalence and covariates associated with having angina among primary care patients is important for several reasons. First, PCPs are the primary source of care continuity for most patients with known and unknown CAD and are, therefore, ideally positioned to screen for and identify underrecognized and undertreated angina. Second, effective, inexpensive treatments for angina are readily available. Third, systematic efforts to ascertain the prevalence and severity of angina in primary care populations may assist PCPs in risk-stratifying patients on the basis of risk of major adverse cardiovascular events and identifying patients who warrant additional testing or evaluation, including referral to cardiology.^12^ In populations with angina prevalence approaching or exceeding that observed here, population-level screening for angina among patients with CAD, coupled with protocols for initiating and uptitrating anti-anginal regimens, represents 1 approach for identifying symptomatic patients and standardizing symptom management. The SAQ-7, which can be administered via email, patient portals, tablet devices in physicians’ offices, mail, telephone, and other modalities, is available in multiple languages and can be used to assess symptoms longitudinally and document treatment response. PCPs could oversee this work, with support from cardiologists when needed.

Consistent with prior work,^7,26,27^ we found that male sex and older age were associated with lower odds of angina, whereas current and prior smoking were associated with higher risk for angina. Associations between angina frequency and atrial fibrillation or peripheral arterial disease have not been extensively studied. Our finding that atrial fibrillation was associated with greater angina frequency could reflect the possibility that less-than-adequate control of ventricular rates during periods of atrial fibrillation predisposes to angina. Given that NOACs are substantially more expensive than coumadin, it is possible that the lower frequency of angina among NOAC users represents residual confounding due to socioeconomic status.^28^ However, additional research is needed to confirm and understand the mechanisms underlying these observed associations.

Previous randomized trials^29,30^ involving patient-reported health outcomes, including angina severity, have failed to demonstrate a clinical benefit associated with using these instruments to track symptoms or inform treatment decisions. This study’s 38.9% response rate, despite the use of dedicated, trained personnel supporting survey administration, underscores the challenges associated with gathering patient-reported health data.

### Limitations

This study has limitations. First, we only evaluated angina frequency at a single time point and cannot draw conclusions about angina patterns over time. Second, our findings may not be generalizable to populations outside our large and demographically diverse health system in the northeastern US. Third, although we had access to all records from the health system where participants received primary care, we lacked access to EHRs for services rendered outside this system and may be missing small amounts of laboratory and/or diagnosis data for select patients. Fourth, our findings may not be generalizable to excluded populations, including patients living in nursing homes and patients who cannot complete a telephone-based survey administered in English or Spanish. Fifth, cardiology consultations are readily accessible in this health system, and cardiology comanagement, for which we did not control, could have influenced angina frequency in a subset of patients. Because cardiology comanagement is unlikely to worsen angina symptoms, our prevalence estimates may represent a lower bound of angina prevalence in primary care populations with higher barriers to accessing cardiologists.

## Conclusions

In conclusion, in this survey study of angina prevalence among stable outpatients with CAD who receive primary care through a large integrated primary care network, 21.2% of surveyed patients reported experiencing angina at least once monthly. Several demographic and clinical characteristics were associated with angina frequency. Systematic evaluation of angina burden using validated assessment tools and prospective estimation of angina burden may improve angina treatment and may be associated with reduced morbidity.

## Notes

Supplement. eMethods. **eTable 1. **Final Disposition Status for Individuals Identified as Being Potentially Eligible for Study Inclusion **eTable 2.** Characteristics of Respondents vs Nonrespondents eReferences.
