# Supplementary material for: Prevalence of Angina Among Primary Care Patients With Coronary Artery Disease
Source: JAMA Netw Open. 2021 Jun 7;4(6):e2112800. doi: 10.1001/jamanetworkopen.2021.12800 (PMC8185599; doi:10.1001/jamanetworkopen.2021.12800)
Supplement: Supplement. — eMethods. eTable 1. Final Disposition Status for Individuals Identified as Being Potentially Eligible for Study Inclusion eTable 2. Characteristics of Respondents vs Nonrespondents eReferences. [file jamanetwopen-e2112800-s001.pdf]

Supplemental Online Content

Blumenthal DM, Howard SE, Searl Como J, et al. Prevalence of angina among primary care patients with coronary artery disease. *JAMA Netw Open*. 2021;4(6):e2112800. doi:10.1001/jamanetworkopen.2021.12800

eMethods.
eTable 1. Final Disposition Status for Individuals Identified as Being Potentially Eligible for Study Inclusion
eTable 2. Characteristics of Respondents vs Nonrespondents
eReferences.

This supplemental material has been provided by the authors to give readers additional information about their work.

© 2021 Blumenthal DM et al. *JAMA Network Open.*

eMethods

1: The Seattle Angina Questionnaire (SAQ) and Seattle Angina Questionnaire-7 (SAQ-7)

SAQ scores have been shown to accurately predict the benefits derived from various approaches to treating symptomatic CAD—including both coronary revascularization and medical management—and has been used to identify racial, ethnic, and gender-related disparities in care quality and delivery.[1] This instrument has been validated extensively and is frequently used to assess angina burden in randomized controlled trials. The "SAQ-7" was developed to address this limitation and includes questions that directly evaluate three of the five domains included in the full length SAQ: Physical Limitation, Angina Frequency, and Quality of Life. The SAQ-7 and SAQ have demonstrated equivalent construct validity, reproducibility, responsiveness to changes in clinical status (e.g. to changes in angina symptoms following PCI), and predictive validity (e.g. the ability to predict 12-month mortality or hospitalization with ACS).[1]

The Seattle Angina Questionnaire-7

1. The following is a list of activities that people often do during the week. Although for some people with several medical problems it is difficult to determine what it is that limits them, please go over the activities listed below and indicate how much limitation you have had **due to chest pain, chest tightness or angina** over the past 4 weeks.

Place an X in one box on each line.

**Activity**	**Extremely limited**	**Quite a bit limited**	**Moderately Limited**	**Slightly limited**	**Not at all limited**	**Limited for other reasons or did not do the activity**
a. Walking indoors on level ground	☐	☐	☐	☐	☐	☐
b. Gardening, vacuuming or carrying groceries	☐	☐	☐	☐	☐	☐
c. Lifting or moving heavy objects (e.g. furniture, children)	☐	☐	☐	☐	☐	☐

2. Over the past 4 weeks, on average, how many times have you had **chest pain, chest tightness or angina**?

I have had **chest pain, chest tightness or angina**...

4 or more times per day	1-3 times per day	3 or more times per week but not every day	1-2 times per week	Less than once a week	None over the past 4 weeks
☐	☐	☐	☐	☐	☐

3. Over the past 4 weeks, on average, how many times have you had to take nitroglycerin (nitroglycerin tablets or spray) for your **chest pain, chest tightness or angina**?

I have taken nitroglycerin...

4 or more times per day	1-3 times per day	3 or more times per week but not every day	1-2 times per week	Less than once a week	None over the past 4 weeks
☐	☐	☐	☐	☐	☐

4. Over the past 4 weeks, how much has your **chest pain, chest tightness or angina** limited your enjoyment of life?

It has **extremely** limited my enjoyment of life	It has limited my enjoyment of life **quite a bit**	It has **moderately** limited my enjoyment of life	It has **slightly** limited my enjoyment of life	It has **not** limited my enjoyment of life at all
☐	☐	☐	☐	☐

5. If you had to spend the rest of your life with your **chest pain, chest tightness or angina** the way it is right now, how would you feel about this?

Not satisfied at all	Mostly dissatisfied	Somewhat satisfied	Mostly satisfied	Completely satisfied
☐	☐	☐	☐	☐

Chan et al.

© 2021 Blumenthal DM et al. *JAMA Network Open.*

2: Coronary Artery Disease Criteria and Algorithm Review (Post-Epic)

Background: Patients with Coronary Artery Disease (CAD) within the larger MGH primary care population represent a subgroup of those with cardiovascular disease that are an important chronic disease population for clinical research, quality assessment, and disease management interventions. This document describes the development and validation of an algorithm to identify patients with CAD with a degree of accuracy in terms of sensitivity (identifying all who have the condition) and specificity (not identifying those without the condition). Algorithms with high positive predictive values help ensure that reporting on CAD quality measures to individual physicians and practices accurately reflects their patients who have this diagnosis. Using an algorithm with a high degree of accuracy will also support efforts to efficiently target patients with CAD for interventions to improve outcomes of care.

The original definition of CAD was developed in 2007-2008. Revisions include:

- November 2010: revision with updated definitions and procedure codes. This included adding ICD-9 billing diagnosis codes and updated search terms to further improve the sensitivity of the algorithm.
- October 2015: added new ICD-10 diagnosis codes.
- November 2016: With the implementation of the EPIC electronic health record in primary care starting on January 28th, 2016, the algorithm was updated to reflect the changes in documentation of patient problem list diagnoses in EPIC.

1. **<u>PCOI Coronary Artery Disease Criteria</u>**:
   a. Inclusion - Adult patients (18 and older) with the following:
      1) One CPT-4 procedure code or procedure list term for CAD intervention ***OR***
      2) Two outpatient visits with 2 problem list terms or 1 ICD billing diagnosis plus 1 problem list term for CAD
   b. Exclusion-None

2. **<u>Data Sources</u>**: (look back time frames)
   a. Pre- EPIC Implementation:
      1) Billing Procedure – CPT (All history)
      2) LMR problem list – key word (All history)
      3) LMR Procedure list – key word (3 years)
      4) Oncall problem and procedure list – key word (3 years)
      5) Billing diagnosis – ICD9/ICD10 (3 years)

   b. Post-EPIC Implementation:
      1) EPIC problem list –ICD9/ICD10 (3 years)
      2) EPIC procedure list – CPT (3 years)

3. **<u>Inclusion Codes and Terms</u>**:

   a. Problem list terms (from LMR and OnCall):

Coronary arteriosclerosis

© 2021 Blumenthal DM et al. *JAMA Network Open.*

Coronary atherosclerosis<br>Coronary artery disease<br>CAD<br>Asymptomatic coronary artery disease<br>Arteriosclerotic heart disease<br>Arteriosclerosis of coronary artery bypass graft<br>Acute coronary occlusion without Myocardial infarction<br>Atherosclerosis of coronary artery bypass graft<br>Atherosclerosis of arterial coronary artery bypass graft<br>Coronary occlusion<br>Coronary bypass<br>Coronary stenting<br>Complication of coronary artery bypass graft<br>Post percutaneous transluminal coronary angioplasty<br>Myocardial infarction<br>Myocardial ischemia<br>Acute subendocardial infarction

b. **Procedure List Terms** (from LMR, OnCall and EPIC):

Coronary artery bypass<br>Coronary artery bypass graft(s)
Cardiac bypass graft surgery
Percutaneous transluminal coronary angioplasty<br>Percutaneous coronary intervention<br>PTCA
Placement of stent in coronary artery
Coronary stent
Cardiac catheterization with stent
Cardiac stent
Coronary stent
CABG
S/P Placement of stent in coronary artery

c. **ICD9 diagnosis codes:**

410.00	Acute myocardial infarction of anterolateral wall, episode of care unspecified
410.01	Acute myocardial infarction of anterolateral wall, initial episode of care
410.02	Acute myocardial infarction of anterolateral wall, subsequent episode of care
410.10	Acute myocardial infarction of other anterior wall, episode of care unspecified
410.11	Acute myocardial infarction of other anterior wall, initial episode of care

© 2021 Blumenthal DM et al. *JAMA Network Open.*

410.12	Acute myocardial infarction of other anterior wall, subsequent episode of care
410.20	Acute myocardial infarction of inferolateral wall, episode of care unspecified
410.21	Acute myocardial infarction of inferolateral wall, initial episode of care
410.22	Acute myocardial infarction of inferolateral wall, subsequent episode of care
410.30	Acute myocardial infarction of inferoposterior wall, episode of care unspecified
410.31	Acute myocardial infarction of inferoposterior wall, initial episode of care
410.32	Acute myocardial infarction of inferoposterior wall, subsequent episode of care
410.40	Acute myocardial infarction of other inferior wall, episode of care unspecified
410.41	Acute myocardial infarction of other inferior wall, initial episode of care
410.42	Acute myocardial infarction of other inferior wall, subsequent episode of care
410.50	Acute myocardial infarction of other lateral wall, episode of care unspecified
410.51	Acute myocardial infarction of other lateral wall, initial episode of care
410.52	Acute myocardial infarction of other lateral wall, subsequent episode of care
410.60	True posterior wall infarction, episode of care unspecified
410.61	True posterior wall infarction, initial episode of care
410.62	True posterior wall infarction, subsequent episode of care
410.70	Subendocardial infarct, episode of care unspecified
410.71	Subendocardial infarct, initial episode of care
410.72	Subendocardial infarct, subesquent episode of care
410.80	Acute myocardial infarction of other specified sites, episode of care unspecified
410.81	Acute myocardial infarction of other specified sites, initial episode of care
410.82	Acute myocardial infarction of other specified sites, subsequent episode of care
410.90	Acute myocardial infarction of unspecified site, episode of care unspecified
410.91	Acute myocardial infarction of unspecified site, initial episode of care
410.92	Acute myocardial infarction of unspecified site, subsequent episode of care
414.00	Coronary atherosclerosis of unspecified type of vessel , native or graft
414.01	Coronary atherosclerosis of native coronary artery
414.02	Coronary atherosclerosis of autologous vein bypass graft
414.03	Coronary atherosclerosis of nonautologous biological bypass graft
414.04	Coronary atherosclerosis of artery bypass graft

© 2021 Blumenthal DM et al. *JAMA Network Open.*

414.05	Coronary atherosclerosis of unspecified artery bypass graft
414.06	Coronary atherosclerosis of native coronary artery of transplanted heart
414.07	Coronary atherosclerosis of bypass graft of transplanted heart
414.2	Chronic total occlusion of coronary artery
414.3	Coronary atherosclerosis due to lipid rich plaque
414.4	Coronary atherosclerosis due to calcified coronary lesion
414.8	Other specified forms of chronic ischemic heart disease
414.9	Chronic ischemic heart disease, unspecified

d. ICD-10 codes (used after 10/1/15)

I21.01	ST elevation (STEMI) myocardial infarction involving left main coronary artery
I21.02	ST elevation (STEMI) myocardial infarction involving left anterior descending coronary artery
I21.09	ST elevation (STEMI) myocardial infarction involving other coronary artery of anterior wall
I21.11	ST elevation (STEMI) myocardial infarction involving right coronary artery
I21.19	ST elevation (STEMI) myocardial infarction involving other coronary artery of inferior wall
I21.21	ST elevation (STEMI) myocardial infarction involving left circumflex coronary artery
I21.29	ST elevation (STEMI) myocardial infarction involving other sites
I21.3	ST elevation (STEMI) myocardial infarction of unspecified site
I21.4	Non-ST elevation (NSTEMI) myocardial infarction
I22.0	Subsequent ST elevation (STEMI) myocardial infarction of anterior wall
I22.1	Subsequent ST elevation (STEMI) myocardial infarction of inferior wall
I22.2	Subsequent non-ST elevation (NSTEMI) myocardial infarction
I22.8	Subsequent ST elevation (STEMI) myocardial infarction of other sites
I22.9	Subsequent ST elevation (STEMI) myocardial infarction of unspecified site
I24.0	Acute coronary thrombosis not resulting in myocardial infarction
I24.1	Dressler's syndrome
I24.8	Other forms of acute ischemic heart disease
I24.9	Acute ischemic heart disease, unspecified
I25.10	Chronic ischemic heart disease without angina pectoris
I25.110	Atherosclerotic heart disease of native coronary artery with unstable angina pectoris
I25.111	Atherosclerotic heart disease of native coronary artery with unstable angina pectoris with documented spasm

© 2021 Blumenthal DM et al. *JAMA Network Open.*

I25.118	Atherosclerotic heart disease of native coronary artery with other forms of angina pectoris
I25.119	Atherosclerotic heart disease of native coronary artery with unspecified angina pectoris
I25.2	Old myocardial infarction
I25.5	Ischemic cardiomyopathy
I25.6	Silent myocardial ischemia
I25.700	Atherosclerosis of coronary artery bypass graft(s), unspecified, with unstable angina pectoris
I25.701	Atherosclerosis of coronary artery bypass graft(s), unspecified, with unstable angina pectoris with documented spasm
I25.708	Atherosclerosis of coronary artery bypass graft(s), unspecified, with other forms of angina pectoris
I25.709	Atherosclerosis of coronary artery bypass graft(s), unspecified, with unspecified angina pectoris
I25.710	Atherosclerosis of autologous vein coronary artery bypass graft(s) with unstable angina pectoris
I25.711	Atherosclerosis of autologous vein coronary artery bypass graft(s) with unstable angina pectoris with documented spasm
I25.718	Atherosclerosis of autologous vein coronary artery bypass graft(s) with other forms of angina pectoris
I25.719	Atherosclerosis of autologous vein coronary artery bypass graft(s) with unspecified angina pectoris
I25.720	Atherosclerosis of autologous artery coronary artery bypass graft(s) with unstable angina pectoris
I25.721	Atherosclerosis of autologous artery coronary artery bypass graft(s) with unstable angina pectoris with documented spasm
I25.728	Atherosclerosis of autologous artery coronary artery bypass graft(s) with other forms of angina pectoris
I25.729	Atherosclerosis of autologous artery coronary artery bypass graft(s) with unspecified angina pectoris
I25.730	Atherosclerosis of non autologous biological coronary artery bypass graft(s) with unstable angina pectoris
I25.810	Atherosclerosis of coronary artery bypass graft(s) without angina pectoris
I25.811	Atherosclerosis of native coronary artery of transplanted heart without angina pectoris
I25.812	Atherosclerosis of bypass graft of coronary artery of transplanted heart without angina pectoris
I25.82	Chronic total occlusion of coronary artery
I25.83	Coronary atherosclerosis due to lipid rich plaque
I25.84	Coronary atherosclerosis due to calcified coronary lesion
I25.89	Other forms of ischemic heart disease
I25.9	Chronic ischemic heart disease, unspecified

© 2021 Blumenthal DM et al. *JAMA Network Open.*

e. **<u>CPT4 Procedure List codes</u>**:

33510:	Coronary artery bypass, vein only; single coronary venous graft
33511:	Coronary artery bypass, vein only; two coronary venous grafts
33512:	Coronary artery bypass, vein only; three coronary venous grafts
33513:	Coronary artery bypass, vein only; four coronary venous grafts
33514:	Coronary artery bypass, vein only five coronary venous grafts
33516:	Coronary artery bypass, vein only; six or more coronary venous grafts
33517:	Coronary artery bypass, using venous graft(s) and arterial graft(s); single vein graft (List separately in addition to code for primary procedure)
33518:	Coronary artery bypass, using venous graft(s) and arterial graft(s) two venous grafts; (List separately in addition to code for primary procedure)
33519:	Coronary artery bypass, using venous graft(s) and arterial graft(s) three venous grafts; (List separately in addition to code for primary procedure)
33521:	Coronary artery bypass, using venous graft(s) and arterial graft(s) four venous grafts; (List separately in addition to code for primary procedure)
33522:	Coronary artery bypass, using venous graft(s) and arterial graft(s) five venous grafts; (List separately in addition to code for primary procedure)
33523:	Coronary artery bypass, using venous graft(s) and arterial graft(s) six or more venous grafts; (List separately in addition to code for primary procedure)
33530:	Reoperation, coronary artery bypass procedure or valve procedure, more than one month after original operation (List separately in addition to code for primary procedure)
33533:	Coronary artery bypass, using arterial graft(s); single arterial graft
33534:	Coronary artery bypass, using arterial graft(s); two arterial grafts
33535:	Coronary artery bypass, using arterial graft(s); three arterial grafts
33536:	Coronary artery bypass, using arterial graft(s); four or more arterial grafts
92980:	Transcatheter placement of an intracoronary stent(s), percutaneous, with or without other therapeutic intervention, any method; single vessel
92981:	Transcatheter placement of an intracoronary stent(s), percutaneous, with or without other therapeutic intervention, any method; each additional vessel (List separately in addition to code for primary procedure)

© 2021 Blumenthal DM et al. *JAMA Network Open.*

92982:	Percutaneous transluminal coronary balloon angioplasty; single vessel
92984:	Percutaneous transluminal coronary balloon angioplasty; each additional vessel (List separately in addition to code for primary procedure)
92975:	Thrombolysis, coronary; by intracoronary infusion, including selective coronary angiography
92977:	Thrombolysis, coronary; by intravenous infusion, including selective coronary angiography

4. **Algorithm Validation**

   a. **Criteria for Chart Review Abstraction to Validate CAD Algorithm**:

      1) **Inclusion**- Adult patients (18 and older) with a diagnosis of CAD within the prior 3 years. Chart review diagnosis is based upon a problem list that includes CAD or related terms (see above list of terms) and a procedure/s that would be appropriate for treating CAD (see procedure code list above). If a patient has a problem list that includes CAD or related terms but no CAD procedure, then the patient was considered to have CAD if the details of the CAD problem list indicated that the patient was considered to have definitive CAD from the provider's perspective. If the patient did not have a problem list that included CAD but had a procedure related to CAD done, it was presumed that the patient has CAD. Other qualifying criteria include PCP or Cardiology notes that discuss CAD status, history or treatment. Individuals treated for CAD in the past, but with stable disease are still considered as having CAD.
      2) **Exclusion**- Patients who do not have a diagnosis of CAD based upon problem list review, or have not had a procedure/s to treat CAD noted.

   b. **Evaluation of Criteria**

      1) **Development**: The process started with a review of our past algorithm, Vascular Disease Documentation-original algorithm2008.doc. We used the original algorithm, which only searched the LMR/Oncall problem/procedure lists to generate an initial CAD population using the 2009 linkage cohort. From this group, a random sample was selected for review. This review identified frequently used search terms for problems and procedures. It also provided a list of non-specific terms that did not define CAD with sufficient specificity (i.e. "stent"). After multiple reviews of this exhaustive list of potential items, a final list of terms that were considered to identify CAD (inclusion terms) and non-specific items (exclusion terms) was agreed upon. A review of original CPT and ICD9 codes compared to updated information resulted in a new list of the most current codes available. To determine whether ICD-9 diagnosis billing codes should be used in addition to EHR

© 2021 Blumenthal DM et al. *JAMA Network Open.*

problems, a comparison of diagnoses based upon problem lists vs. problem lists plus ICD-9 codes was performed. This review found that only 3% of patients without a problem list for CAD had an ICD-9 diagnosis code for CAD. To examine the potential value of these extra patients, a list of 20 randomly selected patients was pulled based on ICD9 codes for CAD from billing data. For the 20 patients with ICD9 codes for CAD, 7 (35%) were found not to have CAD. Given the small increase in cases and the low specificity of the ICD-9 diagnosis code in the absence of a CAD problem, ICD9 codes were not used in the final algorithm to identify patients with CAD. Using this final list of problems and procedure terms from the EHR and CPT codes from billing data, the algorithm was updated and MRNs identified as having CAD were pulled for review.

2) **Validation**: Using the new list of inclusion and exclusion terms and new codes, two lists were pulled from the 2009 PCOI linkage cohort: 1) a list of 15 patients from each practice with CAD based upon the new algorithm, and 2) a list of 10 patients per practice without CAD based upon the new algorithm. A chart review of these 325 patients (13 practices with 25 patients per practice) was performed to validate the algorithm's designation of coronary artery disease status or not. Overall, the results of the review for the search terms and CPT codes demonstrated excellent sensitivity and specificity (Sensitivity =96%, Specificity= 99%, PPV= 99%, and NPV= 95 %.)

3) 2014 Update: In 2012-2013, our vascular disease algorithms were introduced into the Top Care population management tool. As more and more clinicians used Top Care, it was found that the sensitivity of our algorithm, especially PVD needed to be more sensitive. When our original algorithms were developed, we did not include ICD-9 billing or diagnosis codes. Looking to improve the sensitivity of all three algorithms, we added ICD-9 codes as well as any new problem or procedure terms to our search. As with our previous validations, we pulled 15 patients per practice with one or more of our vascular disease categories (CAD, CVD, PVD), and 10 patients per practice with no vascular disease. A blinded cohort of 936 patients was reviewed for validation. After validation we found that for CAD, the addition of the codes did not improve our sensitivity but did not decrease it. For both CVD and PVD, the sensitivity of the algorithm increased.

4) **2015 Update:** In October 2015, ICD-10 codes were introduced to replace ICD-9 for diagnosis billing. We updated our algorithms to reflect this change. No formal validation was done at this time. We did use the new version of the algorithms to compare the numbers from the ICD-9 version with the ICD-10 version. The numbers between the 2014 and 2015 linkage cohorts were comparable, so no formal validation was done at that time.

5) **2017 Update- (post EPIC-Implementation)**: In January of 2016 the institution adopted the EPIC electronic medical record. Prior to EPIC, problem list terms were generated separately from ICD-9/10 diagnostic billing

© 2021 Blumenthal DM et al. *JAMA Network Open.*

terms. In EPIC, problem list terms and diagnostic billing codes are generated as part of the same process. This led to the need to change the algorithm to reflect this lack of independence in choosing problem list terms and billing codes as part of a clinical encounter. Procedure code use remains unchanged in the new algorithms. The new inclusion criteria now include two problem list terms from separate outpatient visits or 1 diagnosis billing code plus a Problem list term also from separate outpatient visits. A blinded list of 656 patients was reviewed for formal validation (10 pts per practice with each condition and 7 pts with none of the conditions).

© 2021 Blumenthal DM et al. *JAMA Network Open.*

3: Methods for Ascertaining and Categorizing Final Disposition Status of Potential Study Participants

The 9356 individuals with CAD who were identified using the PBRN as meeting initial study inclusion criteria—i.e. as being "potentially eligible" for SAQ-7 survey administration—were ultimately grouped into one of four disposition statuses listed below, which align with standard definitions of disposition statuses approved by the American Association of Public Opinion Reporting (AAPOR).[2] All incomplete surveys were categorized as break-offs. The survey completion rate was defined as I/ (I+E).

**Disposition**
**Interviews (I) Completed**
**Eligible, Non-Interview (E)**<br>*Personal or Household Refusal and/or break-off*<br>*Message left, did not return message*<br>*No answer*<br>*Hearing impairment or speech difficulties*[1]<br>*Miscellaneous*[2]
**Unknown Eligibility, Non-Interview (U)**<br>*No screen or interview attempted*
**Not Eligible/Excluded (NE)**<br>*Non-working/disconnected number*<br>*Other*[3]

[1]includes individuals with hearing impairment; moderate or severe dementia; speech difficulties
[2]includes the following groups: those who requested follow up at a later date but never completed their survey and those who did not respond to the SAQ-7 when it was administered via secure email (per their preference)
[3] includes individuals who no longer have an MGH-affiliated primary care physician or are hospitalized, in a nursing home, or on hospice.

© 2021 Blumenthal DM et al. *JAMA Network Open.*

4: SAQ-7 Administration and Documentation of Responses

Each population health coordinator (PHC) was provided with a randomly ordered list of eligible patients from their clinic site(s) and instructed to sequentially contact patients on this list. Seven PHCs administered surveys in English, and one PHC administered a Spanish translation of the SAQ-7 to Spanish-speaking patients across clinics and centers. Each week, one study investigator (DMB) performed up to ten surveys at night or on weekends per patient request. PHCs were instructed to perform at least one follow up call for patients who did not answer their phone, or if patients did not return a voice message from a PHC within 24 hours. Patients who preferred to complete the SAQ-7 by secure email were given the option to do so. We set a goal of surveying at least 50 patients from each primary care clinic or health system. PHCs recorded survey responses in a patient's EHR immediately following survey administration. PHCs were asked to separately document the call date and length, the patient's survey completion status, and relevant notes, including plans for follow-up calls, in the excel file containing their patient lists.

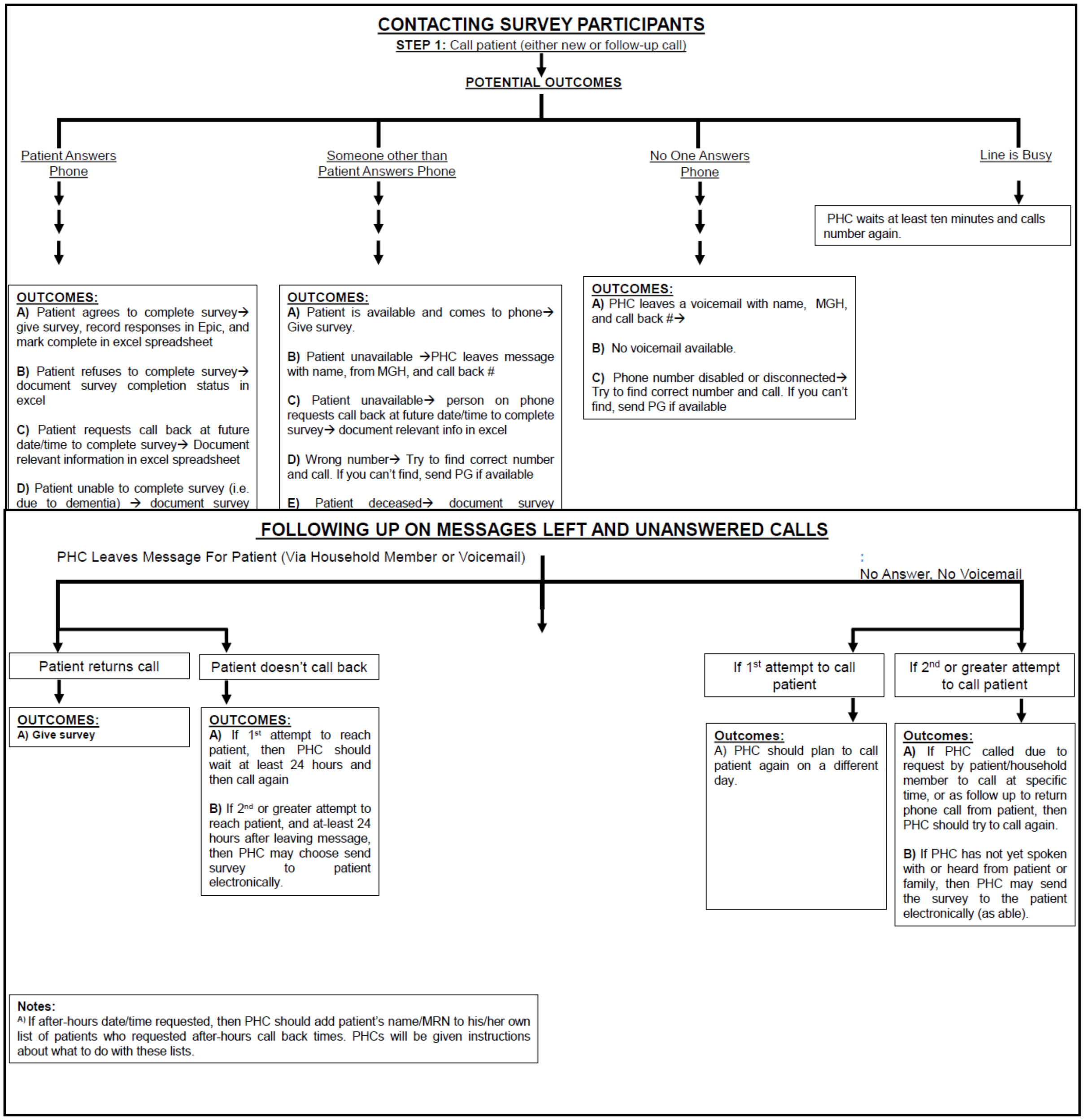

Guidelines for Documenting Phone Calls and Survey Completion Status

For each phone call made to a survey participant, the following information should be documented in the excel spreadsheet:

a. Date of call
b. Approximate length of call
c. Survey completion status using appropriate code in **Figure 1** (either temporary or final, depending on the outcome of the call)
d. Relevant information for follow up plan (e.g. date and time of future call back, plan to send survey to patient via gateway, need for after-hours call by clinician)

FIGURE 1: DOCUMENTING SURVEY COMPLETION STATUS

Survey Completion Status	Corresponding Code
Survey Completed	Y
Participant refused to complete survey	R
Participant unavailable (not home or busy); left message with member of household or on voicemail to have patient return call.	LM
No Answer	NA
Participant unavailable; member of household requested call back at specific date and time ("Future call")	F
Wrong phone number (e.g. outdated or listed incorrectly).	W
Participant unable to complete survey via phone due to:<br>i) Hearing impairment<br>ii) Cant speak<br>iii) Dementia<br>iv) Other (please specify in notes section)	 i) UH<br>ii) US<br>iii) UD<br>iv) UO
Patient deceased	D
Survey sent via Patient Gateway	G
Patient does not have coronary artery disease, or is being surveyed in error	M

Responding to Concerning SAQ-7 Survey Responses

Survey response meets definition for "concerning survey response"

↓

PHC should record responses in EPIC flowsheet per protocol and introduce follow up call per script

↓

PHC should notify on-call clinician about concerning survey response via page. Page should request call back from on-call clinician, and include patient's name, MRN, and phone number, and PHC's phone number.

← On-call clinician doesn't return page within 30 minutes.

↓

Send a second page to on-call clinician.

↓

On-call clinician doesn't return page within 60 minutes.

↓

PHC may page On-call cardiologist (Dr. XXXXX) about concerning survey response. **Once PHC speaks with either of these individuals, PHC is no longer responsible for following up on concerning survey response.**

↓ (from the notification step)

On-call clinician calls PHC to discuss survey response. **On-call clinician becomes responsible for following up with patient.**

© 2021 Blumenthal DM et al. *JAMA Network Open.*

5: Covariate List

I) **Demographic Characteristics:**
   a. mean age
   b. sex
   c. primary language
   d. race/ethnicity
   e. median household income by zip code
   f. insurance type
   g. education level
   h. primary care clinic affiliation

II) **Clinical Characteristics:**
   a. Smoking status
   b. Most recent vital signs
   c. Low density lipoprotein (LDL) level
   d. Hemoglobin A1c (HgBA1c)
   e. Creatinine (Cr)
   f. Hypertension
   g. Diabetes mellitus
   h. Congestive heart failure
   i. Chronic obstructive pulmonary disease
   j. Atrial fibrillation
   k. Prior myocardial infarction
   l. Peripheral artery disease
   m. Dementia
   n. Use of any of the following medications:
      i. Any HMG-Coa reductase inhibitor (i.e. statin)
      ii. Beta blockers
      iii. Non-dihydropyridine calcium channel blockers
      iv. Aspirin
      v. Long acting nitrates
      vi. Short acting nitrates
      vii. Ranolazine
      viii. Any oral $P2Y_{12}$ inhibitor, including clopidogrel, ticagrelor, or prasugrel
      ix. Warfarin
      x. Any novel oral anticoagulant (NOAC), including dabigatran, apixaban, or rivaroxaban
      xi. Insulin

III) **Health care access:**
   a. outpatient clinic appointment frequency for previous three years (2014-2016)
   b. number of missed outpatient clinic appointments over previous three years (2014-2016)

© 2021 Blumenthal DM et al. *JAMA Network Open.*

c. missed appointment ratio over previous three years (2014-2016): The ratio of missed outpatient clinic appointments/ total attended and missed outpatient clinic appointments
d. hospital admission in 2016 (year prior to study start date)

© 2021 Blumenthal DM et al. *JAMA Network Open.*

eTable 1: Final Disposition Status for Individuals Identified as Being Potentially Eligible for Study Inclusion

Disposition	Total Number (N=9356)
**Interview**	**1612**
Complete	*1612*
Incomplete	*0*
**Eligible, Non-Interview**	**2543**
Personal or household refusal and/or break-off	*408*
Message left, did not return message	*888*
No answer and no answering machine	*900*
Hearing impairment or speech difficulties[1]	*81*
Miscellaneous[2]	*250*
**Unknown Eligibility, Non-Interview**	**4567**
No screen or interview attempted	*4567*
**Not Eligible/Excluded**	**650**
Moderate or severe dementia documented in EHR	*53*
Non-working/disconnected number	*44*
Other[3]	*553*

[1]includes the following groups:

1. 77 subjects with hearing impairment
2. 4 subjects with language or speech difficulties preventing survey completion

[2] includes the following groups:

1. 213 subjects requested follow up at a later date but never completed their survey
2. 37 subjects did not respond to the SAQ-7 when it was administered via secure email (per their preference)

[3] includes individuals who no longer have an MGH-affiliated primary care physician or are hospitalized, in a nursing home, or on hospice

© 2021 Blumenthal DM et al. *JAMA Network Open.*

eTABLE 2: Characteristics of Respondents vs Nonrespondents

	**Respondents** (N=1612 unless otherwise noted)	**Nonrespondents** (N=3178 unless otherwise noted)	**Chi[2] or 95% CI**	**P-value[1]**
**DEMOGRAPHIC CHARACTERISTICS**				
**Mean Age—years (SD)**	71.8 (11.0)	73.2 (11.8)	(-2.09, -0.70)	<.001
**Female—% (N)**	35.8 (577)	35.5 (1126)	0.05	0.82
**Primary Language—% (N)[2]**			53.73	<.001
English	89.8 (1447)	90.7 (2881)		
Spanish	9.1 (147)	5.3 (168)		
Other	1.1 (18)	4.0 (128)		
**Race/Ethnicity—% (N)[2]**			12.85	0.005
White or Caucasian	82.8 (1336)	84.1 (2671)		
Black or African American	4.7 (76)	4.6 (147)		
Hispanic or Latino	5.7 (92)	2.6 (114)		
Other	6.7 (108)	7.7 (245)		
**Insurance—% (N)[2]**			7.80	0.051
Commercial	33.8 (545)	32.0 (1017)		
Medicaid	5.2 (83)	4.2 (132)		
Medicare	60.4 (974)	62.6 (1990)		
Self	0.6 (10)	1.2 (38)		
**Median income—% (N)[3]**			1.40	0.67
≤ $40,000	20.4 (329)	20.8 (661)		
$40,001-$80,000	39.5 (637)	40.2 (1276)		
$80,001-$120,000	25.0 (403)	23.5 (746)		
> $120,000	15.1 (243)	15.6 (495)		
**Clinic Affiliation—% (N)[4]**			0.11	0.74
Community Health Center	40.0 (644)	40.5 (1285)		
Not Community Health Center	60.1 (968)	59.6 (1892)		
**Education—% (N)[5]**			6.53	0.163
Eighth Grade or less	5.7 (90)	6.3 (194)		
Some High School	6.3 (99)	6.7 (207)		
High School or GED[6]	33.7 (531)	36.5 (1127)		
Some College, Vocational, or Technical School	10.7 (168)	10.3 (318)		
College Graduate	43.7 (690)	40.2 (1241)		
**CLINICAL COMORBIDITIES**				
**Indication for Study Inclusion— % (N)**			4.63	0.10
Billing claim consistent with prior angina	14.27 (230)	12.21 (388)		
Clinical CAD but no angina	47.52 (766)	47.55 (1511)		
No Clinical CAD	32.21 (616)	40.25 (1279)		

© 2021 Blumenthal DM et al. *JAMA Network Open.*

**Peripheral Vascular Disease—% (N)[2]**	25.3 (407)	22.9 (727)	3.31	0.07
**Cerebrovascular Disease—% (N)[2]**	18.0 (290)	18.6 (592)	0.30	0.58
**Congestive Heart Failure—% (N)[2]**	29.2 (471)	31.6 (1004)	2.85	0.09
**Atrial Fibrillation—% (N)[2]**	26.1 (420)	28.0 (888)	1.94	0.16
**Hypertension—% (N)[2]**	90.3 (1456)	90.2 (2865)	0.02	0.90
**Diabetes Mellitus—% (N)[2]**	39.8 (642)	37.0 (1174)	3.75	0.054
**Previous Acute Myocardial Infarction—% (N)[2]**	12.3 (199)	9.9 (314)	6.77	0.01
**Chronic Obstructive Pulmonary Disease—% (N)[2]**	38.4 (619)	38.5 (1223)	0.004	0.94
**Human Immunodeficiency Virus—% (N)[2]**	0.6 (10)	0.7 (23)	0.17	0.68
**Dementia—% (N)[2]**	4.9 (79)	9.4 (297)	29.24	<.001
**Mean Hemoglobin A1c Among Diabetics—mg/dl (SD)[7]**	6.3 (1.2)	6.4 (1.4)	(-0.17, 0.02)	0.10
**Mean Low Density Lipoprotein Level—mg/dl (SD)[8]**	80 (31)	82 (31)	(-3.78, 0.15)	0.07
**Mean Creatinine—Mg/dl (SD)[9]**	1.14 (0.6)	1.15 (0.7)	(-0.05, 0.02)	0.41
**PRESCRIBED MEDICATIONS**				
**Any Statin—% (N)[2]**	92.5 (1491)	89.4 (2838)	12.13	<.001
**Aspirin—% (N)[2]**	77.4 (1248)	74.2 (2358)	5.88	0.02
**Beta Blocker—% (N)[2]**	78.7 (1269)	77.3 (2455)	1.30	0.26
**Calcium Channel Blocker—% (N)[2]**	9.1 (147)	8.8 (279)	0.15	0.70
**Long-Acting Nitrate—% (N)[2]**	15.7 (253)	13.8 (438)	3.15	0.08
**Short-Acting Nitrate—% (N)[2]**	38.3 (617)	36.3 (1153)	1.81	0.18
**Ranolazine—% (N)[2]**	1.7 (28)	1.4 (45)	0.73	0.39
**UTILIZATION MEASURES**				
**Inpatient Admission in 2016—% (N)[2, 10]**	18.2 (294)	19.9 (631)	1.81	0.18
**Mean number of Outpatient Clinic Appointments, 2014-2016—N (SD)[2, 11]**	19.9 (13)	19.0 (13)	(0.15, 1.70)	0.02
**Mean number of Missed Outpatient Clinic Appointments, 2014-16—N (SD)[12]**	2.2 (2)	2.4 (2.6)	(-0.41, 0.003)	0.054
**Mean missed Appointment ratio—% (SD)[2, 13]**	3.8 (7.1)	5.1 (8.9)	(-1.77, -0.85)	<.001

© 2021 Blumenthal DM et al. *JAMA Network Open.*

NOTES: [1]P-values calculated using T-tests, Chi Square tests, and Mann Whitney U tests as appropriate. [2]Calculated using data for 1612 respondents and 3176 nonrespondents. [3]Median income was estimated using median household income for each patient's zip code. Zip code-level household income data was obtained from the 2010 U.S. Census. [4]Clinic affiliation refers to the clinic where each patient received primary care and were divided into two categories: four community health centers, which are predominately located in lower income, underserved neighborhoods, and 11 non-community health center primary care clinics. [5] Calculated using data for 1578 respondents and 3086 nonrespondents [6]GED: General Educational Development Test. [7]Calculated using most recent available hemoglobin A1c level for 1171 respondents and 2127 nonrespondents. [8]Calculated using most recent available low-density lipoprotein level 1484 respondents and 2757 nonrespondents. [9]Calculated using data on most recent creatinine level from 1574 respondents and 3079 nonrespondents. Patients without a creatinine value in 2015-16 were excluded. [10]The proportion of members with an inpatient admission documented in the electronic health record during 2016. May not include inpatient hospitalizations at facilities outside of Partners Healthcare, the health system which owns the Massachusetts General Hospital. [11]Ascertained using billing and appointment data from Partners Healthcare. Does not include clinic appointments with physicians who aren't affiliated with Partners Healthcare. [12]Ascertained using billing and appointment data from Partners Healthcare for 627 respondents and 1363 nonrespondents. Does not include missed clinic appointments with physicians who aren't affiliated with Partners Healthcare. [13]Calculated in the following way: The ratio of missed outpatient clinic appointments/outpatient clinic appointments was calculated for each patient. Then, the mean and the standard deviation of these ratios were calculated for both respondents and nonrespondents.

eReferences:

1. Chan PS, Jones PG, Arnold SA, Spertus JA. Development and validation of a short version of the Seattle angina questionnaire. *Circ Cardiovasc Qual Outcomes.* 2014;7(5):640-647.
2. American Association for Public Opinion Research. *Standard Definitions: Final Dispositions of Case Codes and Outcome Rates for Surveys.* 2016. 9th Edition. AAPOR.

© 2021 Blumenthal DM et al. *JAMA Network Open.*
